# Does sports participation (including level of performance and previous injury) increase risk of osteoarthritis? A systematic review and meta-analysis

**DOI:** 10.1136/bjsports-2016-096142

**Published:** 2016-09-28

**Authors:** Gui Tran, Toby O Smith, Adam Grice, Sarah R Kingsbury, Paul McCrory, Philip G Conaghan

**Affiliations:** 1 Institute of Rheumatic and Musculoskeletal Medicine, University of Leeds, Leeds, UK; 2 Faculty of Medicine and Health Sciences, University of East Anglia, Norwich Research Park, Norwich, UK; 3 The Florey Institute of Neuroscience and Mental Health, Melbourne Brain Centre—Austin Campus, Heidelberg, Australia; 4 NIHR Leeds Musculoskeletal Biomedical Research Unit, University of Leeds, Leeds, UK; 5 Arthritis Research UK Centre for Sport, Exercise and Osteoarthritis, Nottingham, UK

**Keywords:** Osteoarthritis, Sport, Elite performance, Risk factor, Injury

## Abstract

**Background:**

To assess the relationship between sport and osteoarthritis (OA), and specifically to determine whether previous participation, in terms of level (elite or non-elite), type of sport, intensity or previous injury, was associated with OA.

**Methods:**

This systematic review was developed using PRISMA guidelines. Databases were searched (to May 2016). Narrative review and meta-analysis (with risk ratio (RR) and 95% CIs) approaches were undertaken where appropriate. Study quality was assessed using GRADE.

**Results:**

46 studies were included. Narratively, 31 studies reported an increased risk of OA, with 19 demonstrating an increased risk in elite athletes. There was an increased risk after sports exposure (irrespective of type; RR 1.37; 95% CI 1.14 to 1.64; 21 studies). It remained uncertain whether there was a difference in risk of OA between elite and non-elite athletes (RR 1.37; 95% CI 0.84 to 2.22; 17 studies). The risk was higher in soccer (RR 1.42; 95% CI 1.14 to 1.77; 15 studies) but lower in runners (RR 0.86; 95% CI 0.53 to 1.41; 12 studies). 9 studies showed an association with the intensity of sport undertaken and OA. 5 studies demonstrated a higher prevalence of OA following meniscectomies and anterior cruciate ligament tears. Overall, the evidence was of GRADE ‘very low’ quality.

**Conclusions:**

There was very low-quality evidence to support an increased relationship between sports participation and OA in elite participants. It is unclear whether there is a difference in risk between elite and non-elite participants with further prospective studies needed to evaluate this. Pooled findings suggested that significant injuries were associated with OA in soccer players.

## Introduction

Osteoarthritis (OA) is the most common disease of the synovial joints seen in adults and contributes to a substantial usage of health service resources.^1^ There are multiple risk factors that are implicated in OA including obesity, occupation, muscle weakness, previous injury, nutrition, hormonal factors and potentially sports participation.^1^ Sports participation has been shown to have numerous health benefits by improving metabolic health, bone density, depression, obesity and delaying the onset of chronic diseases.^2^ ^3^ However, its relationship with OA is not fully understood. Given that effective OA therapies are limited, determining the relationship between sports participation and OA is important as it potentially provides a population where interventions may prevent subsequent OA.

There have been several narrative reviews investigating sporting activities and their relationship with OA, with conflicting conclusions.^4–7^ For example, Östör *et al*^6^ determined that elite level running increased the risk of OA, although there was no associated risk in recreational runners. Similarly, Conaghan^5^ and Lequesne *et al*^7^ argued that elite sports participation may increase the risk of OA, and that the risk for runners in particular may be dependent on the rate of miles run. In contrast, Cymet and Sinkov^4^ concluded that there was no increased risk of knee or hip OA in runners.

A systematic review in 2003 demonstrated moderate evidence that sporting activity was associated with hip OA, although OA in other joints was not explored.^8^ In a review of OA and physical activity, Vignon *et al*^9^ found sport to be a risk factor for knee and hip OA, with increased risk associated with both intensity (including duration) of sports participation. These systematic reviews, however, did not investigate individual sports, and nor did the authors investigate other joints except the knee or hips, or the role of previous sporting injuries. Furthermore, there has been no systematic review exploring the relationship between OA and elite sport compared with non-elite sport.

This systematic review aims to examine the relationship between OA and the top 32 most popular English sports by participation,^10^ which represents the majority of the 1.7 million adults who participated in sports. It specifically aims to examine if there is a relationship between previous participation in sports, the level of sports participated in (ie, elite and non-elite sports participation), the intensity of sport and whether previous injury associated with sport is related to OA.

## Methods

### Search strategy

The electronic databases, AMED, CINAHL, MEDLINE, EMBASE and SPORTDiscus, were searched for articles pertaining to the relationship of sports participation and OA. Grey literature and unpublished research and trial registries were also searched. These included the WHO International Clinical Trials Registry Platform, Current Controlled Trials, the US National Institute of Health Trials Registry and OpenGrey (System for Information on Grey Literature in Europe). Searches were performed from database inception to 4 May 2016. The search terms and Boolean operators adopted are presented in box 1. These were modified for each individual database.

Box 1 **Search strategy for MEDLINE search, modified for other search engines**

1. exp Sport/
2. Football [tw]
3. Soccer [tw]
4. Golf [tw]
5. Run*(running OR runner OR cross country OR marathon OR sprint$OR steeplechase)
6. Athletics((track and field) OR javelin OR hammer throw OR hurdles OR long jump OR high jump OR pole vault)
7. Bowls [tw]
8. Tennis [tw]
9. Badminton [tw]
10. Squash [tw]
11. Racquetball [tw]
12. Rugby [tw]
13. Cricket [tw]
14. Equestrian(horse-riding OR dressage.tw. OR show jumping)
15. Hockey [tw]
16. Netball [tw]
17. Basketball [tw]
18. Swimming [tw]
19. Diving [tw]
20. Water polo [tw]
21. Sailing [tw]
22. Angling [tw]
23. Cycling [tw]
24. Boxing [tw]
25. Weightlifting [tw]
26. Snow sports [tw]
27. Skiing [tw]
28. Snowboarding [tw]
29. Skating [tw]
30. Table tennis [tw]
31. Mountaineering [tw]
32. Rowing [tw]
33. Gymnastics [tw]
34. Volleyball [tw]
35. Taekwondo [tw]
36. Rounders [tw]
37. Judo [tw]
38. Fencing [tw]
39. OR/1-38
40. exp Osteoarthritis/
41. Arthrosis [tw]
42. Osteoarthrit*[tw].
43. osteo-arthrit*[tw]
44. osteoarthro*[tw]
45. osteo-arthro*[tw]
46. arthrosis [tw]
47. arthroses [tw]
48. arthrot* [tw]

### Inclusion and exclusion criteria

Studies assessing any relationship between sports and OA were included. Sport was defined as physical activity which is in a competitive environment where rules are adhered to.^11^ This is different from physical activity which was defined as any musculoskeletal force that results in energy expenditure. ‘Exercise’ was defined as planned bouts of physical activity that is structured and for health benefits. Searches were restricted to the top 32 sports based on results from the Active People Survey^10^ in 2013, which provides comprehensive annual statistics on sports participation in England. Only studies which described sports participation were included. Only studies published in English were included. Participants of any age were included in the study. Studies which did not clearly document the diagnosis of OA were excluded. Animal studies were excluded. Review articles, case reports, editorials, letters and comments were excluded.

### Study selection

Searches were performed by three reviewers (GT, AG, TOS). Titles and abstracts from each search result were independently reviewed by the same reviewers against predefined eligibility criteria. Full texts of papers considered as potentially eligible were reviewed independently by the same three reviewers against the eligibility criteria. Studies which met these criteria on full-paper assessment were included in the final review.

### Data extraction and analysis of quality

Data were extracted using a standardised database based on previous systematic reviews.^12–15^ Data were independently extracted by two reviewers (GT, AG). Any disagreement in data extraction was managed through discussion between the reviewers. If consensus could not be reached, a decision was made by a third reviewer (SRK).The Preferred Reporting Items for Systematic reviews and Meta-Analyses (PRISMA) statement checklist and four-phase flow diagram was used to enhance the reporting of this systematic review.

Data extracted included level performed; type of sport participated in; demographics (age, sex, body mass index); sample size; follow-up period; anatomical joints assessed; diagnosis of OA and prevalence of OA. Sports participants were categorised into elite and non-elite categories. The definition of ‘elite’ was subjective since no recognised institution (including the IOC, British Association of Sports and Exercise Medicine, English Institute of Sport or *British Journal of Sports Medicine*) has defined this across sports. For the purposes of this review, ‘elite’ was defined as either professional sporting participation (eg, professional soccer) or sporting activity at national or international level.

Methodological quality of the included studies was assessed using a modified version of van Rijn *et al*'s^14^ appraisal tool. The modifications included a criterion on the definition of the sports sample ability/participation and a definition on OA and how it was diagnosed. Seven criteria were defined: sports sample ability/participation adequately defined; a non-sporting comparative group was used; age-matched controls; a prospective design was used; total number of sports participants in the study >100; clearly diagnosed OA with explanations (radiological, both radiological and clinical or surgery); that the study provided raw data percentages, OR, risk ratio (RR) or estimated RR; and clearly explained statistical significance and their meaning in any conclusions. Each criterion was rated positive, negative or non-applicable. If a particular criterion was not applicable for that study, a positive score was allocated for that criterion. A total score for the methodological quality of each study was calculated by summing the number of positive criteria (range 0–7). Studies with five or more positive criteria were considered to be of ‘high quality’.^14^

Each assessment was undertaken independently by two reviewers (GT, AG). Any disagreements regarding the appraisal score were addressed through discussion. If agreement could not be reached, a decision was adjudicated by a third reviewer (SRK).

### Data synthesis

Study heterogeneity was assessed by inspecting the data extraction tables and original source papers. When evidence of population heterogeneity between studies was evident (eg, age, gender, sporting activity, duration of follow-up analysis, method of diagnosing OA), data were narratively reviewed. When there was a low risk of population heterogeneity in respect of these variables, an unadjusted risk ratio (RR) with 95% CIs was calculated using a Mantel-Haenzel summary estimate. Statistical heterogeneity was assessed with I^2^ and χ^2^ values. Where statistical heterogeneity was evident (I^2^ ≥30%; χ^2^: p<0.10), a random-effects model was adopted. When this did not occur, a fixed-effect model was adopted (Cochrane Handbook).^16^ All statistical analyses were performed on RevMan (Review Manager (RevMan) V.5.1. Copenhagen: The Nordic Cochrane Centre, The Cochrane Collaboration, 2011).

The primary analyses undertaken included the prevalence of OA within a general sporting cohort and the RR of experiencing this compared with non-sporting cohorts. Secondary analyses included the risk of experiencing OA between different sports (eg, football, running, tennis) and between elite versus non-elite sports people; whether there is a relationship between intensity of sporting participation and OA; and whether previous injury sustained during sport (most notably for the assessment of elite sports people) was associated with OA.

A sensitivity analysis was undertaken to investigate the origin of statistical heterogeneity when this was evident. Through this, analyses by study design for cohort versus case–control/cross-sectional were undertaken to account for this possible risk of bias. An assessment of the risk of small sample size publication bias was made using a funnel plot based on the primary analysis (ie, relationship between sporting participation and OA).

The research synthesis results were interpreted using the GRADE framework to assess the evidence level based on the sum of the evidence for each individual analysis.^17^ Through this, the quality of evidence was determined by evaluating the study design and evidence for risk of bias (using the results from van Rijn *et al*'s^14^ appraisal results), inconsistency of results, indirectness of evidence, imprecision and likelihood of publication bias, magnitude of effect size, dose–response, confounders or evidence of spurious effects.^17^

## Results

### Search results

**Figure 1:**
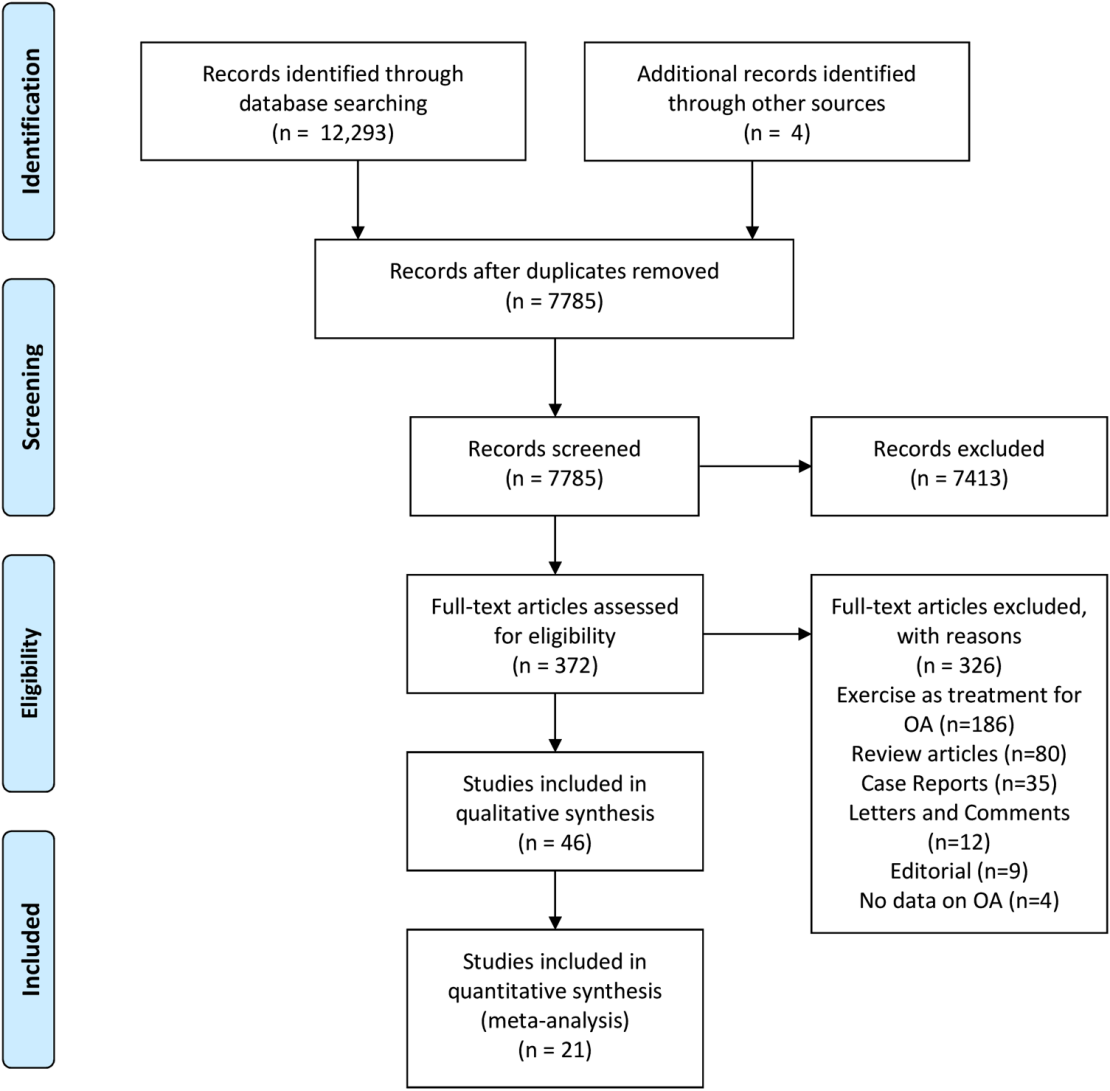
PRISMA flow chart. OA, osteoarthritis.

The results of the search strategy are summarised in the PRISMA flow chart (figure 1). The search strategy identified 12 297 citations. From these, 372 were deemed potentially eligible. After reviewing the titles and abstracts, 46 were deemed eligible and included in the full review.

### Included studies

Characteristics of the included studies are summarised in online supplementary table S1. This included 25 case–control studies (55.3%),^18–42^ 11 cohort studies (23.9%)^43–53^ and 10 cross-sectional studies (21.7%).^54–63^

10.1136/bjsports-2016-096142.supp1 Supplementary table

The most common sport studied was soccer in 15 studies (34.6%),^18^ ^21^ ^25^ ^27^ ^33^ ^34^ ^38^ ^40^ ^41^ ^47^ ^56^ ^58–60^ ^63^ 12 recruited cohorts of long-distance runners (26.1%),^19^ ^22–24^ ^37^ ^43–45^ ^48^ ^52^ ^53^ ^55^ 4 recruited track and field athletes,^31^ ^39^ ^51^ ^62^ 1 recruited people who participated in high jump and javelin,^31^ 1 study javelin alone,^62^ and 1 on high jump alone.^39^ One study recruited swimmers,^54^ one tennis^61^ and one study recruited orienteering runners.^46^ The remaining 12 studies (26.1%) recruited cohorts with mixed sporting participation, that is, some participants participated in soccer, basketball, long-distance running, track and field athletics, ice hockey, racket sports, swimming or golf.^20^ ^24^ ^28–30^ ^32^ ^35^ ^36^ ^41^ ^42^ ^49^ ^50^ ^57^ Twenty-three studies recruited elite sports participants.^18^ ^19^ ^23^ ^25^ ^27^ ^28^ ^30^ ^31^ ^33^ ^34^ ^36^ ^39^ ^49–51^ ^56–59^ ^61–63^ The age of participants ranged from 17^54^ to 70 years.^49^ The follow-up period for longitudinal studies ranged from 2^43^ to 55 years.^51^

Studies most frequently assessed single joints (n=28; 60.9%), those being the knee in 15 studies (32.6%),^27^ ^29^ ^32^ ^33^ ^38^ ^40–42^ ^47^ ^48^ ^54–57^ ^60^ the hip in 8 (17.4%),^19^ ^20^ ^23^ ^25^ ^26^ ^31^ ^35^ ^63^ the ankle in 2 (4.3%),^34^ ^39^ while the upper limb was also assessed in 2 studies (4.3%).^61^ ^62^ The cervical spine was assessed in one study (2.2%).^18^ The remaining 18 studies examined multiple joints. Eight assessed the hip and knee,^21^ ^30^ ^37^ ^45^ ^46^ ^49–51^ while 10 assessed combinations of multiple joints including the knee, hip, ankle, lumbar spine, shoulder, elbow, hand and foot.^22^ ^24^ ^28^ ^36^ ^43^ ^44^ ^52^ ^53^ ^58^ ^59^

OA was diagnosed radiographically in 19 studies (41.3%),^18^ ^19^ ^22^ ^23^ ^25^ ^27^ ^31–33^ ^38^ ^40–43^ ^47^ ^48^ ^50^ ^52^ ^61^ and clinically and radiographically in 12 studies (26.1%);^21^ ^24^ ^34^ ^39^ ^44^ ^45^ ^53^ ^55–57^ ^60^ ^62^ 10 studies (21.7%) used a self-reported diagnosis,^28^ ^30^ ^36^ ^37^ ^46^ ^49^ ^51^ ^58^ ^59^ ^63^ and 3 studies (6.5%) diagnosed OA surgically,^26^ ^29^ ^35^ while 2 studies (4.3%) diagnosed OA solely on symptoms.^20^ ^54^

#### Summary of the methodological quality of included studies

Online supplementary table S1 presents the results of the methodological quality assessment of the included studies. Thirty-three studies (71.7%) were deemed of high quality, and 13 (28.3%) as low quality. Of most importance, 10 studies (21.7%) did not clearly define their cohort's level of sporting participation. Eleven studies (23.9%) provided minimal information on their diagnosis of OA.

#### Assessment of publication bias

**Figure 2:**
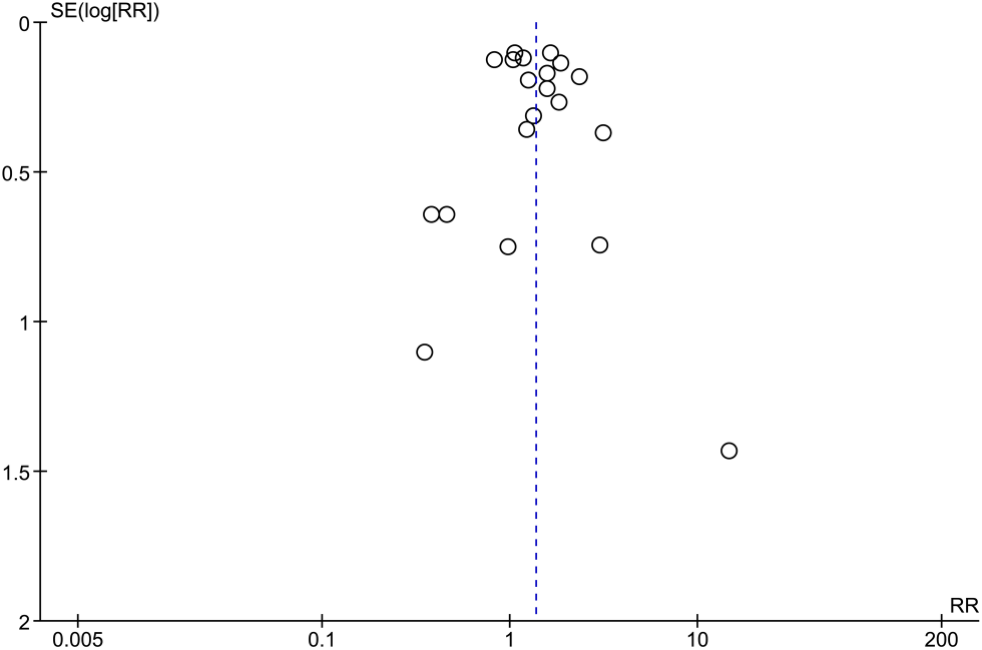
Funnel plot to assess small sample size publication bias. RR, risk ratio; SE(logRR), SE (log risk ratio).

As figure 2 demonstrates, there was a broadly symmetrical funnel plot based on the primary analysis (relationship between sporting participation and OA). Asymmetry suggests that studies are missing and attributed to publication bias.^64^ With a symmetrical funnel plot, the data for this study appeared to be of low risk of small sample size publication bias to impact on this analysis.

### Relationship between sport (non-specific) and OA

Narrative synthesis: Of the 46 studies, 30 (65.2%) reported an increased risk of OA after sports exposure.^18^ ^21^ ^23^ ^25–32^ ^35^ ^36^ ^38^ ^41^ ^42^ ^46^ ^47^ ^49–51^ ^54–60^ ^62^ ^63^ Of these, seven were cross-sectional.^54–60^ The other 16 (34.8%) did not show an increased risk.^19^ ^20^ ^22^ ^24^ ^33^ ^34^ ^37^ ^39^ ^40^ ^43–45^ ^48^ ^52^ ^53^ ^61^

**Figure 3:**
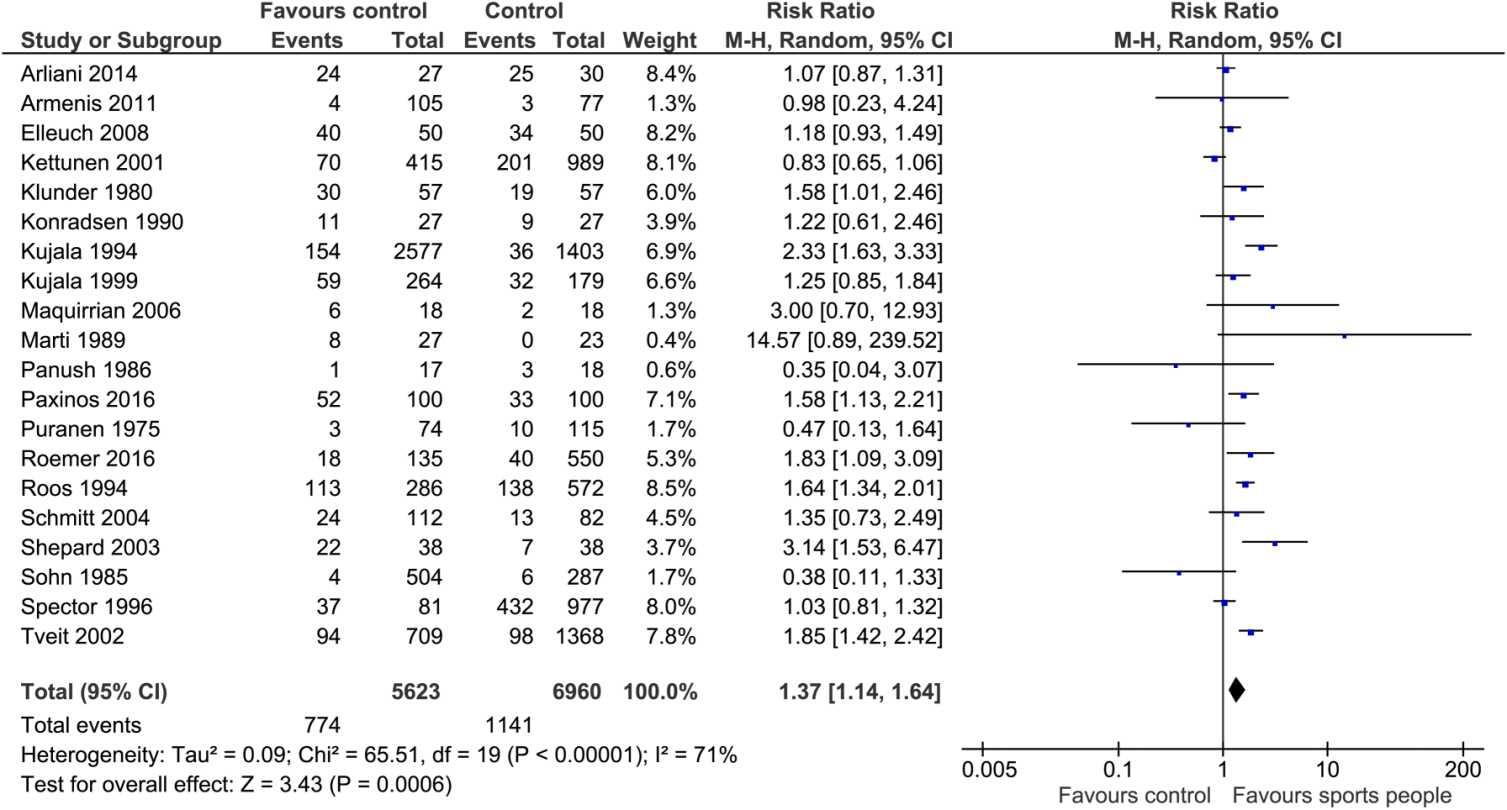
Forest plot assessing the risk of OA after (all) sports exposure in sports people (elite and non-elite) compared with a control group. OA, osteoarthritis.

Meta-analysis: A meta-analysis assessed the relationship in 20 studies based on low risk of study or population heterogeneity. For overall sports participation, there was an increased risk of OA in people who participated in sporting pursuits compared with a control group (RR 1.37; 95% CI 1.14 to 1.64; p<0.01; I^2^=71%; N=12 583; figure 3).

GRADE assessment: The quality of evidence for the relationship between sport (non-specific) and OA was downgraded three levels to ‘very low’ because of study limitations (risk of bias and observational study design), imprecision and inconsistency.

### Relationship between level of sporting participation and OA

Narrative synthesis: Twenty-four studies investigated the relationship between level of sporting participation and OA.^18^ ^23^ ^25^ ^27^ ^28^ ^30^ ^31^ ^36^ ^40^ ^41^ ^49–51^ ^56–59^ ^61–63^ Nineteen (79%) studies of elite sports participants demonstrated an increased risk of OA,^18^ ^23^ ^25^ ^27^ ^28^ ^30^ ^31^ ^36^ ^41^ ^49–51^ ^56–59^ ^61–63^ with one of these demonstrating an increased risk of hospital admission due to OA.^36^ The remaining five studies (20.0%) demonstrated no relationship between sports participation and OA.^19^ ^33^ ^34^ ^39^ ^40^

**Figure 4:**
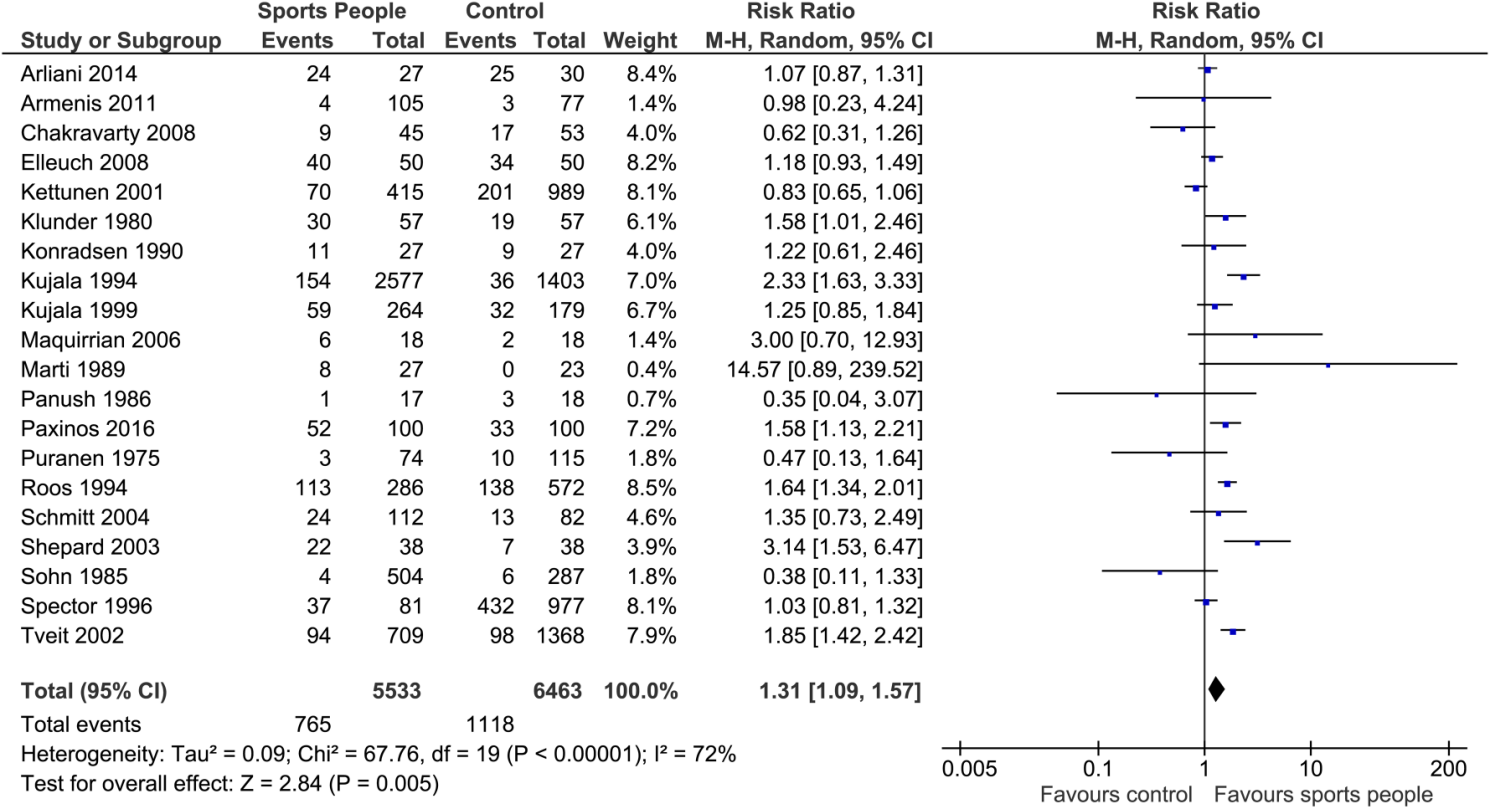
Forest plot assessing the risk of OA after (all) sports exposure in elite sports people compared with a control group. OA, osteoarthritis.

Meta-analysis: When assessed solely for people considered as performing at an elite level (n=20 studies), there was also an increased risk of OA after sports exposure in elite sports people compared with a control group (RR 1.31; 95% CI 1.09 to 1.57; p=0.005; I^2^=72%; N=1384; figure 4).

A sensitivity analysis was undertaken of this analysis only examining cohort study designs. For this evidence base, there was no significant statistical relationship between OA and sports participation in elite sports people compared with non-sports people controls (RR 1.37; 95% CI 0.84 to 2.22; p=0.21; I^2^=88%).

GRADE assessment: The quality of evidence for the relationship between level of sporting participation and OA was downgraded three levels to ‘very low’ because of study limitations (risk of bias and observational study design), imprecision and inconsistency.

### Relationship between type of sport undertaken and OA

Narrative synthesis: In soccer, 12 studies showed a positive relationship between OA and soccer participation.^18^ ^21^ ^25^ ^27^ ^38^ ^41^ ^47^ ^56^ ^58–60^ ^63^ There was no significant relationship between soccer and OA compared with non-sporting participants (teachers, office workers, manual workers and retired participants),^33^ or ankle–foot complex OA.^34^ In one study, there was only a relationship between OA and male soccer players but not in athletics nor tennis players compared with aged/sex-matched controls.^40^

Two papers (16.7%), which recruited long-distance runners alone, demonstrated a relationship between running and hip^23^ and knee OA.^55^ The remaining 10 studies (83.3%) for this sport demonstrated no association.^19^ ^22^ ^24^ ^37^ ^43–45^ ^48^ ^52^ ^53^

Three studies (75%) of the track and field athletic studies involving elite athletes showed a positive association between sports and OA in the elbow^62^ and hip.^31^ ^51^ The other study demonstrated no association with OA in the talotibiofibular joint.^39^

Meta-analysis: It was possible to pool data from 16 papers assessing prevalence of OA in soccer players. This suggested a pooled prevalence of 0.35, the highest prevalence of all sports analysed (table 1). It was possible to estimate the pooled prevalence for a number of other sports. Pooled prevalence of OA in long-distance/middle-distance runners was 0.07 (n=10) and 0.16 (n=8) for track and field athletics. Estimates for this value ranged from 0.19 (n=2) for shooting, to 0.04 (n=2) for tennis.

**Table 1:** Summary of pooled prevalence data assessing the relationship between different sports and osteoarthritis (OA)

Sporting activity	Number of studies	Frequency of OA cases/overall cohort size	Prevalence (95% CI)
Soccer	14	752/2145	0.35 (0.33 to 0.37)
Running	10	81/1135	0.07 (0.06 to 0.09)
Track and field	8	376/2307	0.16 (0.15 to 0.18)
Weightlifting	2	17/142	0.12 (0.08 to 0.18)
Shooting	2	25/135	0.19 (0.13 to 0.26)
Tennis	2	11/248	0.04 (0.03 to 0.08)
Swimming	2	37/253	0.15 (0.11 to 0.20)

There was a statistical relationship between OA and soccer (RR 1.42; 95% CI 1.14 to 1.77; I^2^=66%; p=0.002; see online supplementary figure S1), but no statistical relationship between athletes at elite levels (RR 0.95; 95% CI 0.46 to 1.93; I^2^=96%; N=4; p=0.88; see online supplementary figure S2), or elite long-distance/middle-distance runners (RR 0.86; 95% CI 0.53 to 1.41; I^2^=48%; N=7; p=0.56; see online supplementary figure S3). This finding was also apparent on subgroup analysis comparing elite versus non-elite runners (elite runners (RR 0.91; 95% CI 0.35 to 2.40; I^2^=64%; p=0.85; N=4; see online supplementary figure S4); non-elite runners (RR 0.79; 95% CI 0.49 to 1.28; p=0.34; I^2^=19%; N=3; see online supplementary figure S5)).

10.1136/bjsports-2016-096142.supp2 Supplementary figures

GRADE assessment: The quality of evidence for the relationship between type of sport undertaken and OA was downgraded three levels to ‘very low’ because of study limitations (risk of bias and observational study design), imprecision and inconsistency.

### Relationship between intensity of sporting participation and OA

Narrative synthesis: Ten studies evaluated the relationship between the intensity of sporting participation and OA.^23^ ^26^ ^29^ ^35^ ^37^ ^45^ ^50^ ^54^ ^55^ ^62^ Nine (90%) reported a positive relationship between greater intensity associated with subsequently greater prevalence of OA.^23^ ^26^ ^29^ ^35^ ^45^ ^50^ ^54^ ^55^ ^62^ One study reported no association.^37^

Two studies reported that higher mileage and higher pace was associated with greater subsequent prevalence of OA in the knees^45^ and hip OA in elite athletes.^23^ This relationship was not evident in non-elite athletes as assessed in an additional two studies.^37^ ^55^ In upper limb OA, one study reported an association between 10-year history of using weights >3 kg in training activities and subsequent elbow OA in elite javelin throwers.^62^

Similarly, high exposure to sport was also associated with the increased risk of OA. High exposure was defined by participant recall. This was defined as those in the group with >800 hours exercise in total,^35^ highest quartile of sports activities in the group^29^ and an equally split group classified as having high and medium exposure.^26^ The number of years in combination with long-term ‘vigorous’ exercise increased the risk of OA.^50^ The term ‘vigorous’ was predefined according to sporting activity, which included tennis.

GRADE assessment: The quality of evidence for the relationship between intensity of sporting participation and OA was downgraded two levels to ‘low’ because of study limitations (risk of bias and observational study design) and imprecision.

### Relationship between previous sporting injury and OA

Narrative synthesis: Five articles assessed the relationship between OA and previous sporting injury.^27^ ^38^ ^47^ ^56^ ^60^ One study assessed the relationship between previous meniscectomies and subsequent OA in former soccer players.^27^ The evidence reported a RR of OA being significantly greater than the control (RR=1.64; 95% CI 1.34 to 2.01). This was comparable with the total data set (RR 1.31; 95% CI 1.21 to 1.41).

Two studies reported on the association between OA and soccer players who sustained an ACL injury.^47^ ^60^ Lohmander *et al*^60^ found that 51% had radiographic knee OA. Using multivariate analysis, no significant influence on knee symptoms was found in those who had undergone reconstructive ACL surgery. In a similar study involving male soccer players, 14 years after ACL injury, Kellgren-Lawrence grade 2 or higher changes were seen in 41% of the affected knees compared with 4% of the unaffected knees. On pooled prevalence, OA was more frequently seen in soccer cohorts following ACL injury (prevalence=0.68)^47^ ^60^ compared with the overall soccer cohorts without specific ACL injury (prevalence=0.34; table 1) or for those soccer players postmeniscectomy (0.53).

GRADE assessment: The quality of evidence for the relationship between previous sporting injury and OA was downgraded two levels to ‘low’ because of study limitations (risk of bias and observational study design) and imprecision.

## Discussion

This systematic review has evaluated the evidence on the relationship between participation in the most common sports in England and OA. Across all studies, the relationship between sports participation and OA was equivocal. The results suggested an increased risk of OA for those who participate at the elite level. This is most notably in soccer and athletes, with the exception of long-distance/middle-distance running, compared with non-elite sports participants (p<0.001). The majority of studies in elite sports demonstrated an association between sports participants and risk of OA. In previous reviews, sporting activities have been associated with hip and knee OA, although elite sporting participation has not been separately studied.^5^ ^8^ ^9^ Interestingly, the analyses suggest that the risk of OA may be associated with the type of sport. Indeed, the majority of studies involving soccer demonstrated that there was a positive association with OA, where all the studies involving non-elite soccer players^21^ ^32^ ^38^ ^47^ ^60^ and 80% of studies in elite soccer players^18^ ^25^ ^27^ ^41^ ^56^ ^58^ ^59^ ^63^ demonstrated a positive association. In contrast, both narrative review and meta-analyses indicated no association with OA and running.^19^ ^22^ ^24^ ^37^ ^43–45^ ^48^ ^52^ ^53^ However, using the GRADE approach, the quality of the evidence was assessed as very low quality, meaning that we are very uncertain about the estimates of effect from these findings; thus, the results of the analyses should be viewed with caution.

The results provided some evidence to suggest that higher paced activities and higher exposure to vigorous exercise may increase the risk of OA. Long-distance running has been shown to be associated with OA in long-term sporting activity involving over 90 min of moderate sports participation or 45 min of vigorous exercise^50^ and in athletes who ran >97 km/week and at a faster pace.^23^ Notably, Lane *et al*^43–45^ ^52^ ^53^ only studied between 17.9 and 27 miles ran when no association was found. The association between soccer and OA may also be related to the intensity of sport, as soccer is an activity which lasts over 90 min. These findings were supported in a previous systematic review determining the association between intensity of sport and hip OA.^9^

In an earlier study, more years running but not distance was associated with an increased risk of OA, although no control group was used.^55^ In contrast, no significant difference was found between the risk of OA and the average number of years run or the number of miles run/week in a study using self-reported questionnaires involving varsity runners.^37^

Regarding the risk of previous sporting injuries in the top 32 most popular sports in England^10^ and OA, it was not possible to perform a subgroup analysis to assess this confounding variable on meta-analysis. However, it was possible to assess the point prevalence of different groups. This indicated that those following an ACL rupture presented with the greatest prevalence of tibiofemoral joint OA compared with those following menisectomy and non-injured controls. Overall, this was based on a limited number of studies since there were limited data attributed to those who had an injury compared with those who did not. No trend in the association between previous injury and risk of OA among players who retired through injury was twice that observed among players who had not retired through injury, although this may suggest that the severity of injury was an important confounding factor.

The type of pathologies typically experienced particularly by sporting groups provides an explanation for differences in risk of OA between footballers compared with runners, for example. The injuries sustained during the active professional life of soccer players, and the multidirectional, traumatic injuries associated with soccer, are more typically meniscal lesions and ACL injuries^65^ compared with runners who more frequently have uniplanar, overuse tendinopathy-type injuries.^66^ The intra-articular involvement exhibited in footballers is hypothesised to be a principal reason for higher risk of OA in this population compared with runners which are uniplanter activities and associated with tendinopathy pathologies.

There are several limitations to this review. Imprecision with data gathered, especially with the use of self-reporting questionnaires, exposes studies to high levels of bias. Length of follow-up also varied between studies. The different methods of diagnosing OA by clinical, radiological, surgical or self-reported questionnaires make comparisons difficult. This variability in diagnosing OA would offer different results depending on the methods used, as radiological signs of OA may occur without clinical symptoms.^33^ Furthermore, 26% of studies did not fully explain how they diagnosed OA. This was in part due to the methodology involved in some of the studies, where questionnaires were used to obtain the diagnosis of OA. There has been many in-depth reviews associating injuries and the risk of OA,^67^ ^68^ and it is possible that publications that did not mention sports participation still involved ACL injuries that were a consequence of sporting activity. However, for our review, this did not fulfil our strict inclusion criteria. Furthermore, our focus on the most popular sports in England led to exclusion of studies involving less frequent sports; American Football, for example, was not included. Although a higher level of sport may be related to higher ‘intensity’, many of the articles included did not characterise the ‘intensity’ of sport. For this reason, all the papers investigating intensity were included, regardless of level of sport. There were also relatively few studies investigating OA in the upper extremities compared with the lower extremities.

In conclusion, the relationship between sports participation and OA remains complicated and controversial, being currently based on low-quality or very low-quality evidence. Isolating the effect of sports participation on OA in studies remains difficult. For non-elite participants, the relationship is unclear and further studies need to be conducted with participant sporting ability clearly defined. There is, however, very low-quality evidence supporting a relationship between sports participation and OA in elite participants. Furthermore, a relationship between the intensity of sport undertaken and OA may also exist. The relationship remains to be explored, however, as it is unclear if it is associated with pace, length or duration of training. Although it was not possible to perform a subgroup analysis to assess the risk of previous sporting injury with the risk of OA, increased point prevalence in athletes with previous meniscectomy and ACL rupture has been found.

Future study is warranted to develop knowledge in this field. Research priorities include the assessment of OA in recreational sports people. The evidence base for meta-analysis has been based on elite athletes, and therefore it remains unknown how generalisable these results are to the recreational as well as older sportsperson. Second, a number of important sports were under-represented within the current evidence. Compared with soccer and running, there was limited literature on sports such as rugby, cycling, golf and swimming. These may be available to assess the relationship between OA and OA symptoms in this population. Finally, the majority of studies were based on case–control or cross-sectional study designs. The optimal study design to answer these research questions would be longitudinal cohort studies. These should be adopted for future higher quality studies to further develop understanding in this area.

**What are the findings?**

- There is low-quality or very low-quality evidence to support an increased association of sports participation and the occurrence of osteoarthritis (OA) in elite participants.
- There is very low-quality evidence to suggest that soccer, especially in the elite setting, may increase the risk of OA, whereas running may not.
- For non-elite participants, the relationship is unclear and further prospective cohort studies need to be undertaken.
- Overall, there were conflicting results, based on low-quality evidence, in determining an association between previous sporting injuries and OA, although pooled findings suggest that ACL injuries and meniscectomies may contribute to OA in soccer players.

**How might it impact on clinical practice in the future?**

- Improve awareness that there may be an increased risk of OA in elite athletes, particularly soccer players, and those who get injured.
- This may influence prehabilitation and rehabilitation of these athletes.
- Be aware that high-intensity sporting activity may potentially be associated with OA, although further research needs to be undertaken to understand this.
- Understand that this needs to be balanced with the substantial benefits of participating in sports (physical and mental well-being) where >30 min of activity/day is advised.
