# Supplementary material for: Does sports participation (including level of performance and previous injury) increase risk of osteoarthritis? A systematic review and meta-analysis
Source: Br J Sports Med. 2016 Sep 28;50(23):1459–66. doi: 10.1136/bjsports-2016-096142 (PMC5136708; doi:10.1136/bjsports-2016-096142)
Supplement: Supplementary table [file bjsports-2016-096142supp_table.pdf]

Study	Study design	Methods	Sport	Characteristics of Sports Population	Number of OA cases/ controls<br><br>Sex, age and BMI	Follow-up	Joint Region Studied	Definition of OA	Findings	Quality Score
Sortland [18]	Case control	Twelve of the players regarded themselves as "headers".<br><br>The players have on average participated in 26 international games and in 326 games in Norwegian First and Second division.<br>Clinical and XR undertaken	Soccer	Former elite national soccer team	43/43 (controls were 'different occupational groups)<br><br>Mean age 49.8, range 30-69<br><br>Sex and BMI not mentioned	n/a	Cervical	R	Early development of degenerative changes in the cervical spine in soccer players compared to controls<br><br>Higher frequency and a more marked degree of the degenerative changes in the soccer players	High
Puranen [19]	Case control	Previous Finnish Champions who had hip XR between 1963 – 1974<br><br>Questionnaire sent out at 1973, with 60 replies	Long distance running	Each subject won several Finnish Championships	74/115<br><br>Sex of cases not mentioned; controls were males<br><br>Mean age 55, range 31-81years<br><br>BMI not measured	n/a	Hips	R	No significant difference in hip OA between runners and controls.	Low
Kraus [20]	Case control	First 100 patients from 1974 to 1975 referred to physicians in Sacramen area of University of California with severe advanced OA of hip included.	Athletics NOS	High school athletics participation	100<br><br>Sex collected but not shown<br><br>Mean age 68.4, range 5-89 years<br><br>Data on height and weight obtained and formed into	n/a	Hip	C	No association between OA and participation of high school athletics	Low

					'relative weight groups', where weight: 1.20 or more from 'desirable' or <1.20 from desirable					
Klunder [21]	Case control	Radiographs of the hip and knee joints were taken and a clinical examination<br><br>Soccer players randomly selected at Vejle club	Soccer	Former active soccer players in Denmark<br><br>The average number of playing hours per week in the period of activity was 6.7, and the average period of activity was 22.8 years	57/57<br><br>Males only<br><br>Mean age 56.4, range 40-79 years<br><br>Average weight 78.4 kg	n/a	Hips, knee	B	Osteoarthritis of the hip joint was found to occur significantly more often in the soccer players than in the controls. Such a relationship could not be found regarding the knee joint	High
Panush [22]	Case control	Participants filled in questionnaire, underwent clinical exam and XR of hips, knees and feet.<br><br>Runners aged 50 and over who ran minimum of 20 miles weekly for 5 consecutive years were recruited.	Long Distance Running	Running subjects ran a mean of 44.8 km (28 miles)/wk for 12 years)	17/18<br><br>Males only<br><br>Mean age 56, range 50-74<br><br>Average weight 73.0kg	n/a	Hip, Knee, Ankle	R	Long-duration, high-mileage running need not be associated with premature degenerative joint disease in the lower extremities	High
Marti [23]	Case control		Long distance running	Complete national Swiss Team where runner ran mean of 97km/week	27 runners/23 controls<br><br>Sex not mentioned<br><br>Mean age 42<br><br>BMI not measured	n/a	Hip	R	Long term regular, vigorous, and high mileage long distance running was found to be a significant predictor of degenerative hip<br><br>early radiological signs of degenerative hip disease were found in runners	High

									with extremely high mileage	
Konradsen [24]	Case control	Questions regarding pain and injuries; clinical examination and XR of hips, knees and ankles<br><br>All Danish male orienteering runners who qualified for county teams in the years from 1950 to 1955 were included	Long distance running	County team runners 12-14miles/week for median 40 years	27/27<br><br>Males only<br><br>Mean age 58, range 50- 68 years<br><br>Average weight 71 kg	n/a	Hip, Knee, Ankle	B	Long distance running at mileage levels comparable to those of recreational runners today is not associated with premature osteoarthrosis in the joints of the lower extremities	High
Lindberg [25]	Case control	Former male soccer players representing 10 different soccer clubs in Malmo recruited Elite players recruited from top 2 divisions.<br><br>All had been playing soccer to at least the age of 25<br><br>XR of hips from 1950 - 1988 available for one fifth of patients	Soccer	Former male soccer player in Sweden (71 players elite)	286/572<br>71 elite players<br><br>Males only<br><br>Mean age 55, range 40-88 years<br><br>BMI not measured	n/a	Hip	R	Increased risk of developing hip OA in elite players and in former soccer players.	High
Vingard 1993 [26]	Case-control	Patients receiving hip replacement for idiopathic hip OA recruited from October 1984 to June 1988<br><br>Eligible and willing participants answered telephone interview and questionnaire	Track & Field Athletics Racket Sports	Sports exposure of men who had prosthesis for OA	233/253<br><br>Males only<br><br>Age range 50- 70 year olds<br><br>BMI collected but not shown	n/a	Hip	Surgery	Long-term and high exposure to strenuous sports activities seems to be a risk factor for developing severe OA of the hip	High

Roos [27]	Case control	Patients recruited from Soccer clubs in Malmö > 40 years old who has been active players until at least 25 years old.<br><br>XR of knees reviewed in blinded fashion	Soccer	Former male soccer player in Sweden (71 players elite)	215 non-elite<br>71 elite<br><br>572 aged match male controls<br><br>Mean age 55.6, elite = mean 62.7, non-elite = 53.2; range 40-88<br><br>BMI not measured	n/a	Knee	R	The elite players did not have significantly more meniscectomies than non-elite players but they had a higher prevalence of OA.<br><br>After excluding subjects with known knee injuries, there was no difference between non-elite players and controls, but a higher rate of OA was found among the elite players	High
Sarna [28]	Case control	Athletes representing Finland between 1920-1965 were followed up in 1985<br><br>Subjects traced through records of residence in local parishes<br><br>Records of medications, morbidity and mortality obtained via registries	Soccer<br>Track & Field<br>Ice Hockey<br>Basketball	Athletes who had represented Finland in at least one International Competition	2613/1712<br><br>Males only<br><br>Mean age not documented<br>Age range 20 -65 years<br><br>BMI not shown	n/a	NR	SR	Increased risk of lower limb OA and hospitilisations due to OA	High
Sandmark [29]	Case control	Patients recruited from Swedish Knee Arthroplasty Registry of patients who underwent knee prosthetic surgery due to tibiofemoral OA<br><br>Eligible and willing patients were sent a questionnaire for sporting activity aged 15 – 50 years	Various including Soccer<br>Track and field<br>Tennis	Sport exposure of participants who had knee prosthesis for tibiofemoral OA	625/548<br><br>325 men; 300 women cases<br><br>Age range 53 – 72 years<br><br>BMI not documented but patients matched	n/a	Knee	Surgery	Relative risk higher for men in soccer and track and field<br><br>No increased risk in women<br><br>Moderate daily general physical activity was not found to be a risk	High

		Patients born between 1921 and 1938 were followed up between 1991 and 1993			to controls				factor.<br><br>High exposure to both sports activities and occupational and leisure time physical workload had an increased relative risk compared to those with low exposure, particularly those currently <65 years old	
Kettunen [30]	Case control	Questionnaire sent in 1995<br><br>Athletes who had participated from 1920-1965 in at least one Olympic games World or European championships, or international competitions included	Team sport (soccer, ice hockey, and basketball players)<br>Track and Field(jumpers, sprinters, hurdlers, middle-distance runners, decathlon athletes)<br>Endurance (long-distance runners, cross-country skiers)	Athletes who had represented Finland in Olympics, World or European Championships	1321/814<br><br>Males only<br><br>Endurance Mean age 68.8, range 50.0–92.0 BMI 24.6<br><br>Track and Field Mean age 64.4, range 49.0–88.0 BMI 25.3<br><br>Team Sport Mean age 61.8, range 48.0–95.0 BMI 26.4	n/a	Hip, Knee	SR	Former elite male endurance and track and field athletes and all athletes combined reported no difference in hip or knee OA than did their control subjects<br><br>Team sport athletes had a higher odds for knee osteoarthritis	High
Schmitt 2004 [31]	Case control	Athletes needed to be aged 40 years or greater and >10 years after retirement (average 19 years)<br><br>Recruited from register from 1972 and 1986,	Javelin	Elite athletes with personal best of ≥70 meters for javelin throwers in the 1970s or ≥ 80 meters for javelin throwers in the 1980s;	19/19<br><br>Males only<br><br>Mean age 52, range 40-59<br><br>Mean BMI 28	n/a	Hip	R	Increased risk of hip OA in elite javelin throwers and high jumpers than in age- and BMI-matched non-athletes	High

		which was kept by the German Athletics Association<br><br>Questionnaires, clinical examination and XR undertaken	High Jump	personal best of ≥2.18 meters for high jumpers	22/22<br><br>Males only<br><br>Mean age 47, range 42-57<br><br>Mean BMI 23					
Thelin [32]	Case control	Patients recruited from x-ray verified femorotibial OA at six hospitals in southern Sweden<br><br>Questionnaires sent: 89% of cases responded to questionnaire	Soccer<br>Athletics<br>Tennis	Population in Sweden	825/825<br><br>356 men; 469 women<br><br>Mean age 62.6, range 51-70<br><br>BMI collected but not shown	n/a	Knee	R	Knee OA was related to soccer After adjusting fro confounding<br><br>The sports-related increased risk for knee OA was explained by knee injuries in soccer	High
Elleuch [33]	Case control	Top-level soccer players, having trained regularly for more than 10 hours a week, over the 10-year period from<br>1970 to 1980 and had stopped at least 20 years before the start<br>of the study<br><br>Questionnaires, knee examination and XR taken	Soccer	Male First Division soccer players inTunisia	50/50<br><br>Males only<br><br>Mean age 49.2 years, range 45 – 55 years,<br><br>Mean BMI 24.9	n/a	Knee	R	No significant difference in radiological knee OA between soccer players and controls	High
Armenis [34]	Case control	Former soccer players >40 years old recruited<br><br>Patients had sporting activity from adolescence to 25 years<br><br>150 questionnaire replies; 105 cases underwent clinical examination; 75 had radiographs	Soccer	Male former elite soccer players	150/132<br><br>Males only<br><br>Mean age 49.8 years, range 42 - 55 years<br><br>BMI 26.3	n/a	Ankle Foot Complex	B	No significant difference between groups in developing OA	High

Vingard 1998 [35]	Case control	Patients with hip OA identified from National Registry of total hip replacements<br><br>Sport exposure for women collected via telephone interview and questionnaire	Various	Population in Sweden	230/273<br><br>Females only<br><br>Age range 50 -80 years<br><br>BMI not shown	n/a	Hip	Surgery	Increased risk of hip OA in those with high and medium sport exposure compared to those with low exposure	High
Kujala 1994 [36]	Case control	Athletes who had represented Finland in international events during 1920-65<br><br>Hospital admissions for osteoarthritis of the hip, knee, and ankle joints identified from the national hospital discharge registry between 1970 and 1990.<br><br>Questionnaire posted in 1985	Various including running, soccer, athletics, power sports (boxing, wrestling, throwing)	Elite international athletes representing Finland in the past	2049/1403<br><br>Males only<br><br>Cases: Endurance sport = 281; soccer 252; track and field 498; power sport 770<br><br>Endurance = mean age= 50.5, range 27-82 years, Soccer = mean 45.4, range 23-76 years, Track and Field = mean 45.6, range 24-80 years, Power sport = mean 47.0, range 23-85 years old<br><br>BM calculated but not shown	n/a	Hip, knee, ankle	SR	Athletes from all types of competitive sports are at slightly increased risk of requiring hospital care because of osteoarthritis of the hip, knee, or ankle.	High
Sohn [37]	Case control	Subjects were selected from seven major eastern colleges, who competed between 1930 and 1960<br><br>Questionnaires sent<br><br>Mean follow-up 25 years, range 2 – 55 years	Long distance running	Varsity Cross Country Runners from 7 Eastern US Colleges	504/287 (controls were swimmers)<br><br>Sex not shown<br><br>Mean age 57, range 23 - 77<br><br>BMI not shown,	n/a	Hip, Knee	SR	There is no association between moderate long-distance running and development of osteoarthritis.<br><br>Neither heavy mileage nor the	High

					although patients were 'weight' matched with controls				number of years running are contributory to OA	
Neyret [38]	Case control	All patients had rim preserving meniscotomies in France; patients either had intact ACL (G1; 41 knees) or ruptured ACL (G2; 50 knees)<br><br>Patients reviewed retrospectively at two time points: once at 5 years post meniscotomy and second a minimum 20 years, average 27 years<br><br>Patients contacted by mail and asked to fill in questionnaire; underwent clinical examination for instability; XR for OA diagnosis	Soccer	Soccer players who subsequently had menisectomy	77 participants: 41 knees with intact ACL vs 50 knees with ruptured ACL<br><br>Males only<br><br>Average age at injury was 24 years (range, 12 to 41), at operation was 28 years (range, 17 to 44), and at follow-up was 53 years (range, 39 to 67)<br><br>In G2, the average age at injury was 23 years (range, 17 to 32), at operation was 27 years (range, 18 to 40), and at follow-up was 55 (range 41-66)<br><br>BMI not shown	n/a	Knee	R	The rate of osteoarthritis was significantly higher when the ACL was ruptured as diagnosed by radiological changes;	Low
Schmitt 2003 [39]	Case control	Athletes jumping >2.18m recruited from register from 1972 and 1986, which was kept by the German Athletics Association<br><br>>10 years after retirement<br><br>Patients filled in	High Jumpers	Former elite high jumpers 10 years post retirement from competition	40/40<br><br>Males only<br><br>Mean age 41.8, range 32 - 56<br><br>Mean BMI 23.2	n/a	Talotibiofibular ankle joint	B	No significant radiological differences seen on XR	High

		questionnaire and underwent clinical examination and XR								
Arliani [40]	Cross-sectional	Players played > 5years<br><br>History, examination and KOOS recorded	Soccer	Male former 1st division professional soccer	27/30 (non-sports professional areas)<br><br>Mean age 45.67<br><br>All males<br><br>Mean BMI 28.35 years	n/a	Knee	R (radiographs and MRI)	Prevalence of OA in soccer group was 66%.<br><br>Former soccer players have a worse quality of life and greater radiographic changes in radiographic compared with a control group ($p<0.05$)	High
Paxinos [41]	Cross sectional	Players who had played > 5 years recruited<br><br>KOOS and medical/sporting history completed + clinical examination<br><br>Ultrasound for cartilage thickness, osteophytes, effusion	Soccer	Retired professional national soccer championship	100/100 (active duty Hellenic Air Force military personnel)<br><br>All male<br><br>Mean age 46.9 range 35-55 years	n/a	Knee	R (sonography)	Higher prevalence of sonographic OA in soccer players vs control, even when excluding history of knee surgery ($p=0.01$)<br><br>No difference in KOOS	High
Roemer 2015[42]	Cross-sectonal	Patients 18-36 years referred to secondary care	"Athletes" (non-elite); 82% soccer players	Most but not all participants were registered to sports clubs in Qatar	135/550 ("non-athletes")<br><br>81.2% male<br><br>Mean age 28.5 years	n/a	Knee	R	The adjusted OR for OA for athletes were 2.8 (95% CI 1.4, 5.5)	High
Lane 1990 [43]	Prospective cohort	Member recruited from Fifty-Plus Runner's Association<br><br>Patients within 100 miles of Stanford, , invited to	Long Distance Running	Runners aged 50 and over and a member of a running club in the US	34/34<br><br>62% male<br><br>Mean age 59.8, range 50-72 years	2 years	Knee, Hand, Lumbar Spine	R	In the 50-Plus Runners Association group, running did not appear to influence	High

		Stanford for rheumatologic examination and XR<br><br>XR of the hands, lateral lumbar spine, and knees in 1984 and 1986			BMI in 1984 = 22.5<br><br>BMI in 1986 = 22.7				the development of radiologic osteoarthritis; women showed more osteophyte formation in the knee joints	
Lane 1993 [44]	Prospective cohort	Member recruited from Fifty-Plus Runner's Association<br><br>Patients within 100 miles of Stanford, , invited to Stanford for rheumatologic examination and XR<br><br>XR of the hands, lateral lumbar spine, and knees in 1984 and 1989	Long Distance Running	Runners aged 50 and over and a member of a running club in the US	35/38<br><br>Mean age 63.3, range 50-70 years<br><br>Weight in 1984 = 67.0kg<br><br>Wight in 1989 = 67.1kg	5 years	Knee, Hand, Lumbar Spine	B	Running did not accelerate the development of radiographic or clinical OA of knees	High
Lane 1998 [45]	Prospective cohort	Member recruited from Fifty-Plus Runner's Association<br><br>Patients within 100 miles of Stanford, , invited to Stanford for rheumatologic examination and XR<br><br>XR knees in 1984, 1986, 1989, 1993 and XR hips 1993	Long Distance Running	Runners aged 50 and over and a member of a running club in the US	28/27<br><br>17 males; 11 females<br><br>Mean age 66.4 range 60-77 years<br><br>BMI in 1984 = 22.5<br><br>BMI in 1993 = 23.6	9 years	Hip, Knee	B	No accelerated development of knee or hip OA in runners vs non-runners	High
Kujala 1999 [46]	Prospective cohort	Athletes who were placed among the 60 best in their master orienteer age-class in Finland in 1984 recuited<br><br>Questionnaires sent	Orienteering running	Top 60 in age category in Finland	269/188<br><br>Males only<br><br>Mean age 48.6, range 37 – 61 years<br><br>BMI 23.2	11 years	Hip, Knee	SR	Runners reported knee osteoarthritis and knee pain more often than did the controls, whereas the occurrence of hip osteoarthritis and hip pain did not differ	High

von Porat [47]	Retrospective cohort	Swedish soccer players with knee injuries in 1986 identified from insurance company<br><br>Eligible patients filled in questionnaire and underwent XR	Soccer	Male Swedish soccer players with ACL tear identified from insurance company archives in 1986	122/contra-lateral knee as control<br><br>Males only<br><br>Mean age 38, range 30-56<br><br>BMI 26	14 years after the initial injury	Knee	R	High prevalence of knee OA seen in soccer players post knee injury<br><br>No differences were seen between surgically and conservatively treated players.	High
Chakravarty [48]	Prospective cohort	Runners recruited from Fifty-Plus Runners Association<br><br>A geographically convenient sample from the original cohort of all 113 individuals residing within 100 miles of Stanford University was invited to participate in the radiographic longitudinal study	Long distance running	Recreational runners aged over 50 years	45/53<br><br>Cases: 30 males; 15 females<br><br>Mean age 58 years, range 50 – 72 years in 1984<br><br>BMI 22.3 at first radiograph; 23.1 at last radiograph	Serial knee radiographs taken from 1984 to 2002	Knee	R	Long-distance running among healthy older individuals was not associated with accelerated radiographic OA	Low
Tveit [49]	Retrospective cohort	Mailed questionnaires to former national and internationally ranked athletes from Sweden.<br><br>Patients retired from sport median 35 years ago	Various including soccer	Elite athletes: impact (including soccer) and non-impact (including long distance runners and swimmers)	709/1368<br><br>Males only<br><br>Median age 70 years old, range 50-93<br><br>Mean BMI for impact athletes 26.2; non-impact athletes 26.6	Duration of follow up not mentioned	Hip and knee	SR	Increased risk of hip and knee OA in athletes compared to control<br><br>Previous knee injury is associated with knee OA in former impact athletes but not in nonimpact athletes	High
Spector [50]	Retrospective cohort	National or international players between 1950 and 1979 recruited with help from International Athletics Club and Lawn	Long distance Running Tennis	Female elite athletes in the UK	67 middle and long distance runners<br>14 tennis players<br><br>977 age matched	Patients playing between 1950-1979 recruited and invited for radiographs at time of study	Hip, Knee	R	Weight-bearing sports activity in women is associated with increased risk of radiologic OA	High

		Tennis Association<br><br>Patients invited to attend XR hips/knees and clinical exam			controls<br><br>Females<br><br>Mean age 52.3 years, range 40-65<br><br>Mean BMI 22.1	(1996).			Duration rather than frequency of training may be an important contributory factor as there was a similar increased risk in a subgroup from the general population who reported long-term sports activity	
Vingard 1995 [51]	Retrospective cohort	All persons 50-80 years of age were selected from the Swedish Athletic Association registers<br><br>Questionnaire sent	Track & Field Athletics	Elite athletes - winners of International or National Championships for Swedish Athletes	114/355<br><br>Males only<br><br>Age range 50 -80 years<br><br>BMI collected but not shown	Followed up from age of 25 to current study (1995)	Hip, Knee	SR	Increased risk of OA amongst athletes compared to controls	High
Lane 1986 [52]	Retrospective cohort	Member recruited from Fifty-Plus Runner's Association<br><br>Questionnaires sent<br><br>Patients within 100 miles of Stanford, including 41 runners and 57 control subjects, invited to Stanford for rheumatologic examination and XR	Long distance Running	Runners aged 50 and over in the US	41/41<br><br>56% male<br><br>Mean age for 50+ runners = 57.5, range 50-72 years<br><br>Average weight 66.4 kg	n/a	Knee, Hand, Lumbar Spine	R	No association of running and OA found	Low
Lane 1987 [53]	Retrospective cohort	Case-control<br><br>Member recruited from Fifty-Plus Runner's Association<br><br>Patients within 100 miles	Long Distance Running	Runners aged 50 and over and a member of a running club in the US	498/365<br><br>86% male<br><br>Mean age 58.6, range 50-72 years	n/a	Knee, Hand, Lumbar Spine	B	Little evidence for any deleterious effects of long-distance running on development of radiologic	High

		of Stanford, including 41 runners and 57 control subjects, invited to Stanford for rheumatologic examination and XR			Mean BMI 22.8				osteoarthritis,	
Stulberg [54]	Cross-sectional	Patients recruited from swimming clubs in the Chicago area<br><br>All of the swimmers in considered the breaststroke to be their main event	Swimming	Club level Swimmers	23/no controls<br><br>9 men; 14 women<br><br>Average age 17 years, range 6-30 years<br><br>BMI not shown	n/a	Knee	C	Breaststrokers who had been using the whipkick for > 8 years had clinical evidence of patellofemoral osteoarthritis	Low
McDermott [55]	Cross-sectional	Runners who had been competing for at least five years were selected randomly from a group complaining of knee pain for > 3 months.<br><br>Participants asked to fill in questionnaire, including previous injury, and undergo clinical examination and XR	Long distance running	Middle and long distance runners complaining of knee pain	20/no controls<br><br>Males only<br><br>Mean age 38.5, range 29-55 years<br><br>BMI not measured	n/a	Knee	B	OA found in 6 runners<br><br>Oa predicpsoed to degenerative changes<br><br>The number of years spent training was significantly greater in the OA group than in the those without OA	Low
Chantraine [56]	Cross-sectional	Soccer players recruited played 6-15 years 'top' level sport<br><br>Clinical and radiographs taken	Soccer	Former professional soccer players from Switzerland<br><br>Patients had intensive sport for average 19 years, range15 years to 25 years	81/no controls<br><br>Sex not mentioned Average age of 48.8 years, range 40 - 74 years,<br><br>BMI not mentioned	n/a	Knee	B	Increased risk of OA in soccer players compared to control	Low
Kujala 1995 [57]	cross-sectional	Athletes who, between the years 1920 and 1965, represented Finland at least once in the Olympic games, in world or European championships, or	Soccer<br>Long distance running	Elite athletes who had represented Finland in Olympics, World or European Championships	28 runners<br>31 soccer players<br><br>No control<br><br>Males only	n/a	Knee	B	Soccer players are at increased risk of developing premature knee OA. The increased risk is explained in part	Low

		in inter-country competitions<br><br>Questionnaire mailed to surviving athletes in 1985; respondents invited to interview, clinical examination and XR			Soccer = mean age 56.5, range 45-67 year<br>BMI 22.9<br><br>Long distance running = mean age 59.7, range 51-67 years<br>BMI 21.7				by knee injuries	
Turner [58]	Cross-sectional	Mean professional career length was 13.5 years<br><br>Questionnaires sent to five Former Players Associations	Soccer	Former elite professional soccer players from UK	284/no controls<br><br>Sex not mentioned<br><br>Mean age 56.1 years<br><br>BMI not measured	n/a	Knee, hip, ankle, foot	SR	Total of 138 (49%) respondents reported receiving a diagnosis of OA, with knee the most common site<br><br>Playing professional soccer can impact on the health of United Kingdom soccer players in later life	Low
Drawer [59]	Cross-sectional	Questionnaire s sent to 500 former players registered with the English Professional Footballers' Association	Soccer	Former elite professional soccer players from UK	185/no controls<br><br>Sex not mentioned<br><br>Mean age 47.6 years, range 20 – 84 years,<br><br>BMI not mentioned	n/a	Hip, Knee, Ankle	SR	32% (59) had been medically diagnosed with osteoarthritis in at least one of the lower limb joints	Low
Lohmander [60]	Cross-sectional	Female soccer players who sustained an ACL injury 12 years earlier were contacted<br><br>Patients consented to knee XR and questionnaires	Soccer	Female Swedish soccer players with ACL tear identified from insurance company archives in 1986	103 patients<br>No controls<br><br>Females only<br><br>Mean age 31, range 26-40 years	n/a	Knee	R and SR	High prevalence of radiographic knee OA, pain, and functional limitations was observed in young women who sustained an ACL	Low

					Mean BMI 23				tear during soccer play 12 years earlier - (42%) were considered to have symptomatic radiographic knee OA	
Maquirrian [61]	Cross-sectional	Patients older than 50 years, previous professional level and continuous activity were recruited<br><br>Glenohumeral XR taken	Tennis	Former elite professional male tennis players	18/18<br><br>17 males<br><br>Mean age 57.2, range 51-75 years<br><br>BMI not measured<br><br>All players were right handed and they hit one-handed backhand	n/a	Shoulder	R	Prevalence of glenohumeral osteoarthritis in the dominant shoulder of the players was greater in former elite tennis players (33% (95% confidence interval (CI)), 13% to 59%) than in sedentary controls (11% (95% CI, 1% to 34%))<br><br>But overlapping CI between groups, therefore not significant	High
Schmitt 2001 [62]	Cross sectional	Elite athletes at local German institution from 1970s and 1980s examine<br><br>Average of 19 years (range 10 to 25 years) after the end of their 'high performance phase'<br><br>Questionnaire, physical examination undertaken; XR elbows and MRI shoulders done	Javelin	Elite male athletes to include several World-record holders and Olympic gold-medallists	21<br><br>Males only<br><br>Mean age 50, range 35 - 60<br><br>Right handed<br><br>No mean BMI reported, although<br><br>Contralateral shoulder also	n/a	Shoulder, Elbow	B	All dominant elbows had advanced OA changes compared to the non-dominant side<br><br>Athletes who trained with weights of more than 3 kg had a significantly higher risk of	Low

					assessed				degenerative changes than athletes who did not	
Shepard [63]	Cross-sectional	Questionnaires sent to ex professional soccer players who were current managers of soccer league or premiership teams	Soccer	Ex-professional soccer players	74/136<br><br>Sex not documented<br><br>Mean age 44 years, range 32–59<br><br>BMI not documented<br><br>Mean playing career length was 16 years	n/a	Hip	SR	The ex-professional soccer players had a significantly higher prevalence of OA of the hip	High

NOS = Not otherwise specified; NR = Not Reported; R = Radiological; C = Clinical; SR = Self-Reported; B = both; OA = osteoarthritis; XR = X-Ray; BMI = body mass index

Supplementary Table 1: Characteristics of included studies and quality score assessment results
