# Supplementary material for: Does sports participation (including level of performance and previous injury) increase risk of osteoarthritis? A systematic review and meta-analysis
Source: Br J Sports Med. 2016 Sep 28;50(23):1459–66. doi: 10.1136/bjsports-2016-096142 (PMC5136708; doi:10.1136/bjsports-2016-096142)

Supplementary Figure 1: Forest-plot representing the risk ratio of osteoarthritis in soccer players compared to non-sporting controls.

Supplementary Figure 2: Forest-plot representing the risk ratio of osteoarthritis in athletes compared to non-sporting controls.

Supplementary Figure 3: Forest-plot representing the risk ratio of osteoarthritis in runners compared to non-sporting controls.

Supplementary Figure 4: Forest-plot representing the risk ratio of osteoarthritis in elite runners compared to non-sporting controls.

Supplementary Figure 5: Forest-plot representing the risk ratio of osteoarthritis in non-elite runners compared to non-sporting controls.

	Sports People		Control			Risk Ratio
**Study or Subgroup**	**Events**	**Total**	**Events**	**Total**	**Weight**	**M-H, Random, 95% CI**
Arliani 2014	24	27	25	30	19.3%	1.07 [0.87, 1.31]
Armenis 2011	4	105	3	77	2.1%	0.98 [0.23, 4.24]
Elleuch 2008	40	50	34	50	18.4%	1.18 [0.93, 1.49]
Klunder 1980	30	57	19	57	12.0%	1.58 [1.01, 2.46]
Konradsen 1990	11	27	9	27	6.9%	1.22 [0.61, 2.46]
Paxinos 2016	52	100	33	100	15.1%	1.58 [1.13, 2.21]
Roos 1994	113	286	138	572	19.5%	1.64 [1.34, 2.01]
Shepard 2003	22	38	7	38	6.6%	3.14 [1.53, 6.47]
**Total (95% CI)**		**690**		**951**	**100.0%**	**1.42 [1.14, 1.77]**
Total events	296		268			

Heterogeneity: $Tau^2 = 0.05$; $Chi^2 = 20.45$, df = 7 (P = 0.005); $I^2 = 66\%$

Test for overall effect: Z = 3.13 (P = 0.002)

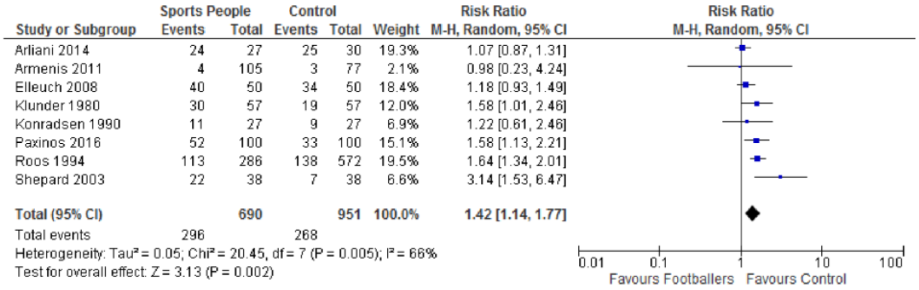

	Sports People		Control			Risk Ratio
**Study or Subgroup**	**Events**	**Total**	**Events**	**Total**	**Weight**	**M-H, Random, 95% CI**
Kettunen 2001	373	1697	201	989	26.6%	1.08 [0.93, 1.26]
Kujala 1994	120	2049	36	1403	25.2%	2.28 [1.58, 3.29]
Schmitt 2004	24	1121	13	82	22.2%	0.14 [0.07, 0.26]
Tveit 2002	94	709	98	1368	26.0%	1.85 [1.42, 2.42]
**Total (95% CI)**		**5576**		**3842**	**100.0%**	**0.95 [0.46, 1.93]**
Total events	611		348			

Heterogeneity: $Tau^2 = 0.49$; $Chi^2 = 69.64$, df = 3 (P < 0.00001); $I^2 = 96\%$
Test for overall effect: Z = 0.15 (P = 0.88)

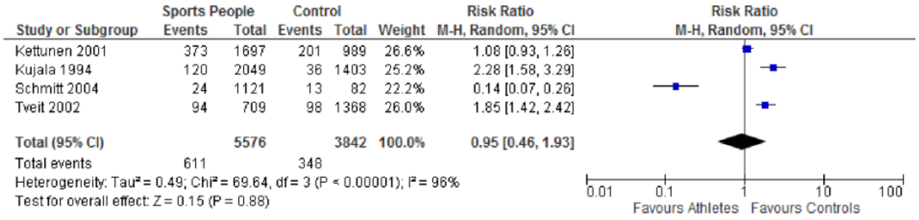

Study or Subgroup	Sports People Events	Sports People Total	Control Events	Control Total	Weight	Risk Ratio M-H, Random, 95% CI
Chakravarty 2008	9	45	17	53	20.8%	0.62 [0.31, 1.26]
Konradsen 1990	11	27	9	27	20.8%	1.22 [0.61, 2.46]
Kujala 1999	59	264	32	179	29.4%	1.25 [0.85, 1.84]
Marti 1989	8	27	0	23	2.9%	14.57 [0.89, 239.52]
Panush 1986	1	17	3	18	4.6%	0.35 [0.04, 3.07]
Puranen 1975	3	74	10	115	10.8%	0.47 [0.13, 1.64]
Sohn 1985	4	504	6	287	10.8%	0.38 [0.11, 1.33]
**Total (95% CI)**		**958**		**702**	**100.0%**	**0.86 [0.53, 1.41]**
Total events	95		77			

Heterogeneity: Tau² = 0.18; Chi² = 11.47, df = 6 (P = 0.07); I² = 48%
Test for overall effect: Z = 0.59 (P = 0.56)

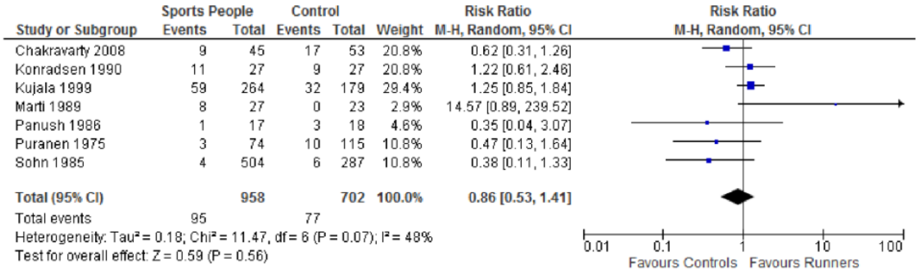

Study or Subgroup	Sports People Events	Sports People Total	Control Events	Control Total	Weight	Risk Ratio M-H, Random, 95% CI	Risk Ratio M-H, Random, 95% CI
Kujala 1999	59	264	32	179	40.5%	1.25 [0.85, 1.84]	
Marti 1989	8	27	0	23	9.4%	14.57 [0.89, 239.52]	
Puranen 1975	3	74	10	115	25.1%	0.47 [0.13, 1.64]	
Sohn 1985	4	504	6	287	25.0%	0.38 [0.11, 1.33]	
**Total (95% CI)**		**869**		**604**	**100.0%**	**0.91 [0.35, 2.40]**	
Total events	74		48				

Heterogeneity: Tau² = 0.56; Chi² = 8.30, df = 3 (P = 0.04); I² = 64%
Test for overall effect: Z = 0.19 (P = 0.85)

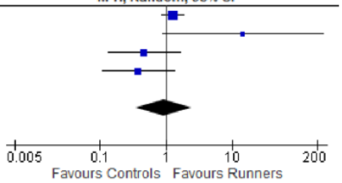

Study or Subgroup	Sports People Events	Sports People Total	Control Events	Control Total	Weight	Risk Ratio M-H, Random, 95% CI
Chakravarty 2008	9	45	17	53	46.6%	0.62 [0.31, 1.26]
Konradsen 1990	11	27	9	27	46.8%	1.22 [0.61, 2.46]
Panush 1986	1	17	3	18	6.6%	0.35 [0.04, 3.07]
**Total (95% CI)**		**89**		**98**	**100.0%**	**0.82 [0.47, 1.45]**
Total events	21		29			

Heterogeneity: Tau² = 0.05; Chi² = 2.45, df = 2 (P = 0.29); I² = 19%
Test for overall effect: Z = 0.67 (P = 0.50)

Risk Ratio
M-H, Random, 95% CI

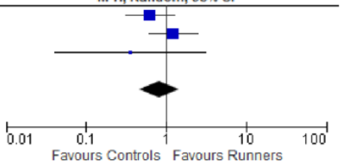

Supplement: Supplementary figures [file bjsports-2016-096142supp_figures.pdf]
